# Targeting Mechanotransduction at the Transcriptional Level: YAP and BRD4 Are Novel Therapeutic Targets for the Reversal of Liver Fibrosis

**DOI:** 10.3389/fphar.2016.00462

**Published:** 2016-12-01

**Authors:** Altynbek Zhubanchaliyev, Aibar Temirbekuly, Kuralay Kongrtay, Leah C. Wanshura, Jeannette Kunz

**Affiliations:** ^1^Department of Biology, School of Science and Technology, Nazarbayev UniversityAstana, Kazakhstan; ^2^Department of Biotechnology and Microbiology, Faculty of Natural Sciences, L.N.Gumilyov Eurasian National UniversityAstana, Kazakhstan; ^3^BioScribiaLafayette, CO, USA

**Keywords:** fibrosis, myofibroblasts, mechanotransduction, Hippo pathway, YAP, bromodomain, BRD4

## Abstract

Liver fibrosis is the result of a deregulated wound healing process characterized by the excessive deposition of extracellular matrix. Hepatic stellate cells (HSCs), which are activated in response to liver injury, are the major source of extracellular matrix and drive the wound healing process. However, chronic liver damage leads to perpetual HSC activation, progressive formation of pathological scar tissue and ultimately, cirrhosis and organ failure. HSC activation is triggered largely in response to mechanosignaling from the microenvironment, which induces a profibrotic nuclear transcription program that promotes HSC proliferation and extracellular matrix secretion thereby setting up a positive feedback loop leading to matrix stiffening and self-sustained, pathological, HSC activation. Despite the significant progress in our understanding of liver fibrosis, the molecular mechanisms through which the extracellular matrix promotes HSC activation are not well understood and no effective therapies have been approved to date that can target this early, reversible, stage in liver fibrosis. Several new lines of investigation now provide important insight into this area of study and identify two nuclear targets whose inhibition has the potential of reversing liver fibrosis by interfering with HSC activation: Yes-associated protein (YAP), a transcriptional co-activator and effector of the mechanosensitive Hippo pathway, and bromodomain-containing protein 4 (BRD4), an epigenetic regulator of gene expression. YAP and BRD4 activity is induced in response to mechanical stimulation of HSCs and each protein independently controls waves of early gene expression necessary for HSC activation. Significantly, inhibition of either protein can revert the chronic activation of HSCs and impede pathological progression of liver fibrosis in clinically relevant model systems. In this review we will discuss the roles of these nuclear co-activators in HSC activation, their mechanism of action in the fibrotic process in the liver and other organs, and the potential of targeting their activity with small molecule drugs for fibrosis reversal.

## Introduction

Chronic fibroproliferative disease is a major cause of death in the industrialized world, placing a substantial burden on healthcare systems (Mehal et al., 2011; Rockey et al., 2015). In the liver, chronic injury from a variety of etiologies, including hepatitis, obesity/metabolic syndrome, and inflammation, leads to liver fibrosis (Ballestri et al., 2016). While fibrosis due to hepatitis can be treated with antiviral therapies, nonviral sources remain difficult to treat (Poordad et al., 2014; Zeuzem et al., 2014) and once progressed, treatment is limited to slowing escalation of the disease, but cannot reverse liver fibrosis (Friedman et al., 2013). The only treatment option for end-stage liver disease to date is liver transplantation; but this is limited by availability of donor organs and associated with significant long-term complications and costs. While the late stages of liver fibrosis are irreversible, mounting evidence from relevant rodent models and some patient studies suggest that the early stages of fibrosis and, potentially, even cirrhosis are reversible (Liu et al., 2013). Thus, there is an urgent need for the development of more effective therapeutic strategies that aim to target liver fibrosis and better knowledge of the molecular events underlying the early stages of fibrosis holds the key to blocking disease progression.

Regardless of tissue type and etiology, fibrosis is the result of an aberrant wound healing response to tissue injury or inflammation, characterized by excessive remodeling of the extracellular matrix and progressive scarring (Mehal et al., 2011; Rockey et al., 2015). This wound healing response is largely driven by mechanotransduction—that is, intracellular signaling that is initiated by and mediated through mechanical forces generated by physical interactions between cells and the extracellular microenvironment. During the chronic fibrotic response, mechanical signaling initiated by extracellular matrix proteins, such as interstitial collagens, leads to a positive feedback loop by which fibrotic cells produce an excess of extracellular matrix proteins. This, in turn, leads to increased tissue stiffness and further activation of fibrotic cells. In the liver, this process is mainly mediated by HSCs that become activated and differentiate into myofibroblasts that are proliferative and fibrogenic (Iwaisako et al., 2014). The switch from quiescent HSC to myofibroblast involves mechanotransduction-induced gene transcription of fibrillary collagens and alpha smooth muscle actin (α-SMA), leading to stress fiber formation, extracellular matrix deposition and increased cell-extracellular matrix contact. Ultimately, the resulting tissue contraction promotes chronic HSC activation through a positive feedback loop involving the microenvironment and HSCs that perpetuates HSC activation (Hinz et al., 2007; Friedman, 2008; Butcher et al., 2009; Mederacke et al., 2013). Importantly, while fibrogenic signaling is activated during the earliest disease stages, extracellular matrix accumulation leading to fibrotic scar development only occurs during the course of chronic tissue injury. Uncovering the molecular mechanisms that drive early steps in HSC activation and sustain the deposition of extracellular matrix therefore is key to halting the mechanosensitive feedback loop and reversing the fibrotic response.

Recent studies suggest that the gene transcription program necessary for HSC activation is controlled, at least in part, by two transcriptional regulators: Yes-associated protein (YAP) and bromodomain-containing protein 4 (BRD4) (Ding et al., 2015; Mannaerts et al., 2015). Although these transcriptional regulators appear to function independently of each other through unique molecular mechanisms during stellate cell activation, each is regulated by the microenvironment during liver fibrosis. Importantly, pharmacologic inhibition of YAP or BRD4 with small molecules that disrupt their respective interactions with cofactors has produced promising antifibrotic responses in murine liver fibrosis models (Ding et al., 2015; Mannaerts et al., 2015) and thus, represents an attractive therapeutic approach to treat liver fibrosis in patients. In this review, we will discuss the roles of these transcription factors in fibroproliferative diseases, their potential contribution to mechanotransduction during HSC activation, and the potential of targeting these proteins that mediate extracellular matrix stiffness-induced gene expression to treat patients with fibrosis.

## Hepatocellular stellate cells mediate liver fibrosis

Liver fibrosis is mediated by myofibroblasts that in mice arise from two cell types: HSCs are the major source of myofibroblasts in hepatocellular injury (carbon tetrachloride; CCl_4_) induced fibrosis, whereas portal fibroblasts are the major source of myofibroblasts in response to cholestatic injury (bile duct ligation) induced fibrosis (Iwaisako et al., 2014). Interestingly, HSCs become activated by portal fibroblasts with longer durations (14–20 days) of bile duct ligation-induced cholestatic liver injury, thereafter promoting chronic fibrosis. These cholestatic injury-induced HSCs are “portal fibroblast-like,” possessing a gene expression profile—including Thy1 and elastin—similar to portal fibroblast-derived myofibroblasts and distinct from that of hepatocellular injury-induced myofibroblasts, which are characterized by Vitamin A and glial fibrillary acidic protein (GFAP) expression (Mederacke et al., 2013; Iwaisako et al., 2014). Thus, HSC activation ultimately represents the major source of chronic liver fibrosis in both hepatocellular and cholestatic models.

**Figure 1:**
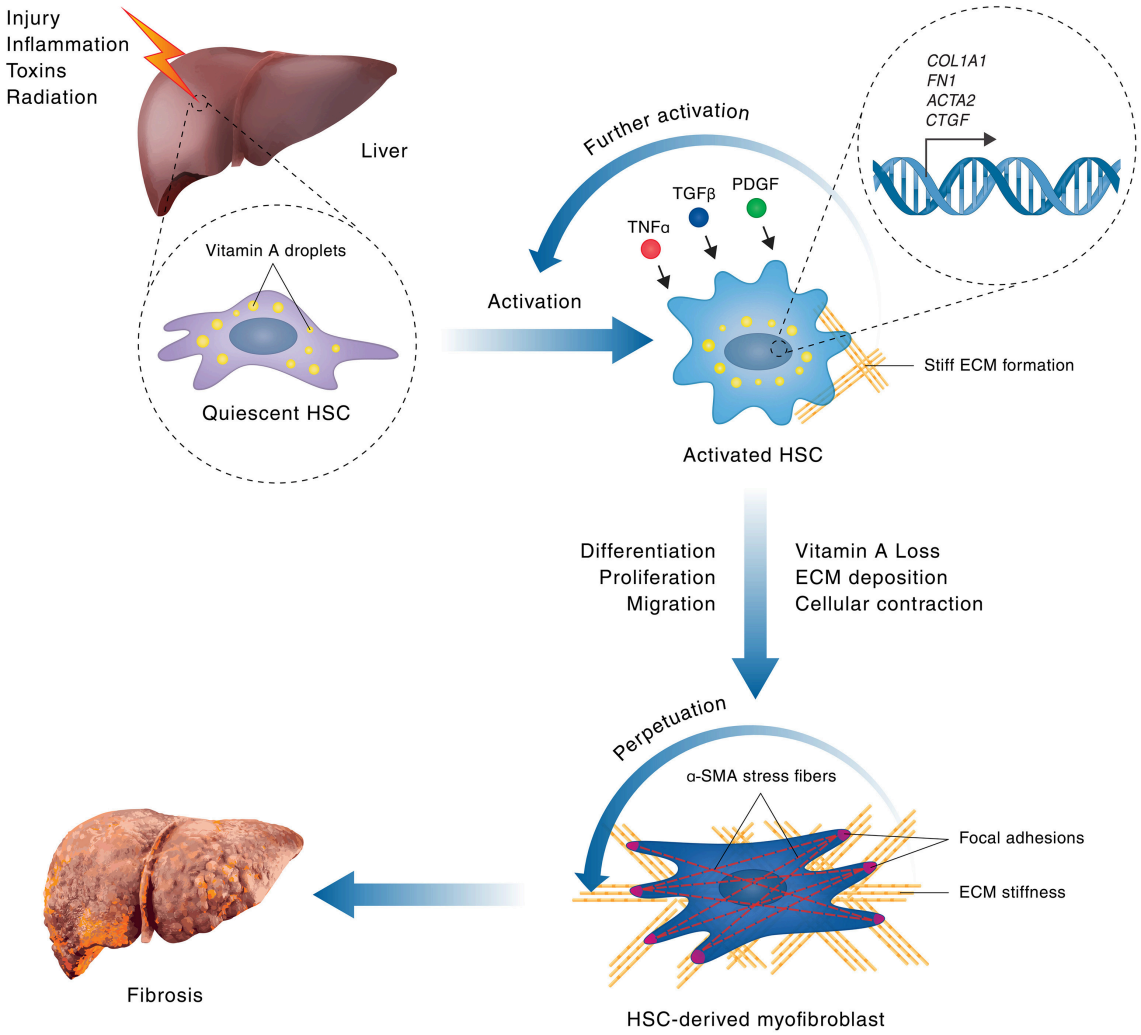
**Hepatic stellate cell activation in liver fibrosis**. A schematic model of HSC activation in fibrosis is shown. Hepatocellular injuries or inflammation lead to activation of quiescent, vitamin A-rich, HSCs via signaling processes that involve cytokines, such as TNF-α, TGF-β1 and PDGF. HSC activation leads to upregulation of gene expression of genes encoding pro-proliferative, cytoskeletal and extracellular matrix components, such as *Acta2* (encoding αSMA), *Col1A1, FN1* (encoding Fibronectin 1), and *Ctgf*, leading to subsequent stimulation of HSC proliferation and extracellular matrix secretion. During chronic liver injury, a positive feedback loop of self-sustained, pathological HSC activation is stimulated where increased HSC proliferation leads to excessive deposition of extracellular matrix. The resulting increase in matrix stiffness promotes further stress fiber and focal adhesion formation in HSCs by mechanotransduction, leading to increased contractility, thus perpetuating HSC activation and promoting their transformation into proliferative, fibrogenic myofibroblasts, which are the main mediators of fibrosis. The persistent population of myofibroblasts, and the resultant crosslinked collagen matrix they produce, causes progressive formation of fibrous scar tissue, ultimately leading to the destruction of normal tissue architecture and the development of liver fibrosis.

In healthy livers, quiescent, vitamin A-rich HSCs reside in the space of Disse (Iwaisako et al., 2014). In response to liver injury (Figure 1), HSCs proliferate and deposit extracellular matrix components such as interstitial collagens and matrix metalloproteinases (MMPs) (Friedman, 2008; Mederacke et al., 2013). HSCs are thereby ultimately converted into proliferative, fibrogenic myofibroblasts that express α-SMA (Hinz et al., 2007; Friedman, 2008). HSC-derived myofibroblasts upregulate matrix deposition, including fibrillary types I, III, and IV collagen that are characteristic of all fibrotic diseases and are each increased up to 10-fold in advanced liver fibrosis (Westergren-Thorsson et al., 1993; Lucey et al., 1998; Schuppan et al., 2001; Gressner and Weiskirchen, 2006; Hinz et al., 2007; Kisseleva and Brenner, 2008). Upon liver damage, HSC-derived myofibroblasts proliferate and migrate to the site of injury to promote wound healing by depositing and remodeling extracellular matrix components that encapsulate the injury (Figure 1). During chronic liver injury, however, aberrant wound healing leads to hyperproliferation and reduced apoptosis of myofibroblasts. Increased contact between myofibroblasts and the extracellular matrix is mediated by α-SMA expression-induced stress fiber and focal adhesion (FA) formation. This increased contact leads to increased contractility, inducing mechanotransduction-related signaling (Butcher et al., 2009) and resulting in excessive extracellular matrix component deposition, cross-linking of fibrous scar tissue, and increased tissue stiffness characteristic of human fibroproliferative disease (Figure 1). Through autocrine and paracrine mechanisms, extracellular matrix components such as MMPs, fibrillary collagens and proteoglycans promote growth factor (mainly PDGF) and cytokine signaling. This signaling, particularly through the transforming growth factor-beta (TGF-β) and Wnt pathways, further activates HSCs, thus creating a positive, profibrotic feedback loop (Figure 1, Karsdal et al., 2015; Li et al., 2015). Ultimately, mechanotransduction- and growth factor-induced gene regulation by transcriptional regulators such as YAP and BRD4 mediate fibroproliferative disease and represents a unique target for pharmacological intervention for patients with liver fibrosis.

## YAP-induced mechanotransduction promotes liver fibrosis

**Figure 2:**
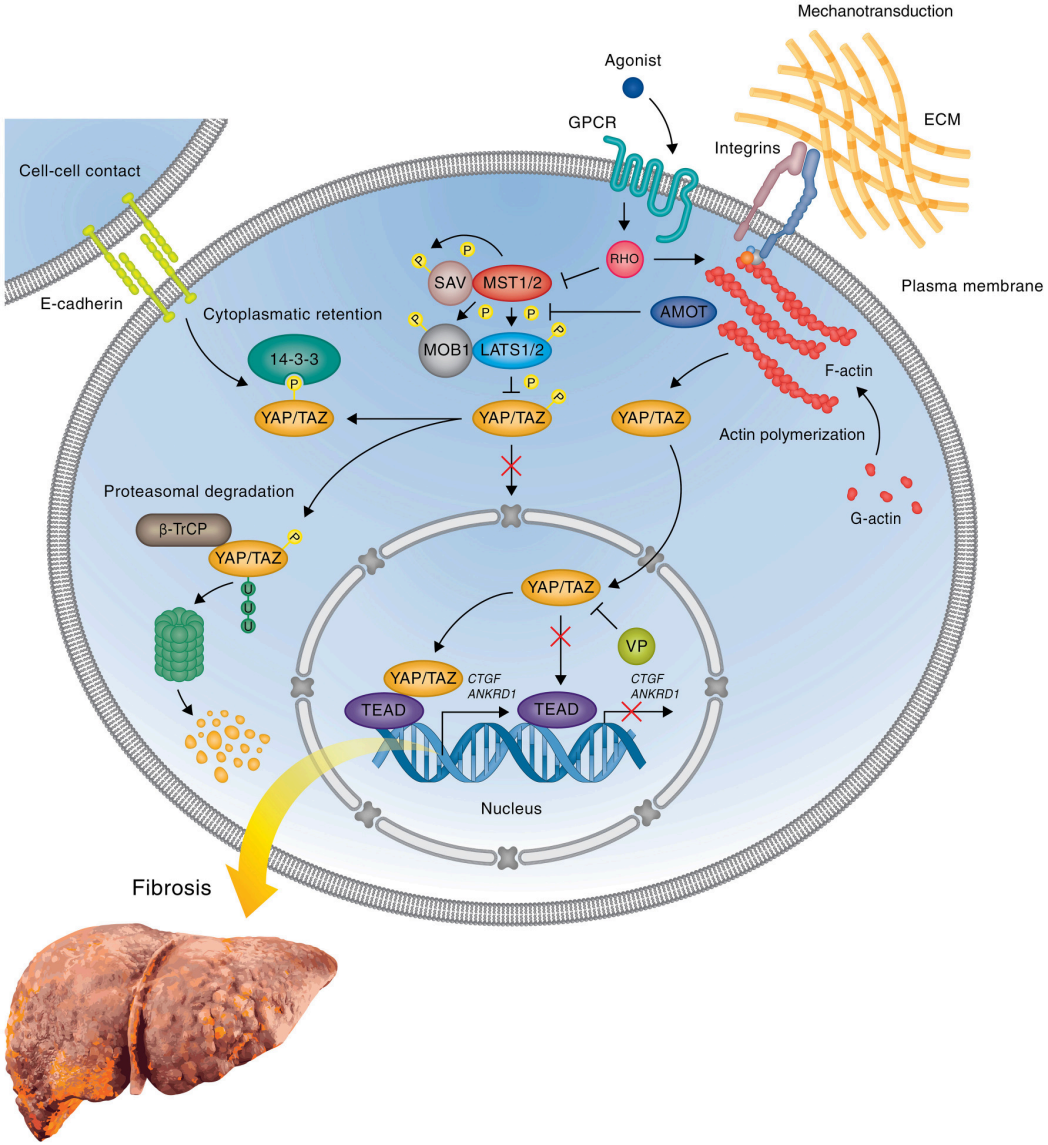
**YAP mediated gene expression in liver fibrosis**. Simplified scheme showing the regulation of profibrotic gene expression by YAP during liver fibrosis and attenuation of gene expression by the inhibitor VP. When the Hippo pathway is on, the active Hippo core kinase complex phosphorylates YAP on five serine residues leading to either the cytoplasmic sequestration of YAP through interaction with 14-3-3 proteins or the degradation of YAP by the proteasome. Inactivation of the Hippo core kinase complex in response to initial tissue injury or subsequent mechanotransduction abrogates YAP phosphorylation, leading to nuclear translocation of YAP, complex formation with TEAD transcription factor(s), and upregulation of profibrotic genes, such as *Ctgf* and *Ankrd1*. In cells treated with the FDA-approved drug VP, which blocks the interaction between YAP and TEADs, YAP/TEAD mediated profibrotic gene expression is blocked, thus preventing HSC activation and inhibiting or reversing the development of fibrosis. The precise mechanism leading to the activation of Hippo pathway activity in response to upstream signals during liver fibrosis remains to be determined. Potential upstream regulators including cell-cell contact, GPCRs, and integrins are shown.

YAP and the homologous transcriptional co-activator with PDZ-binding motif (TAZ) are the ultimate effectors of the Hippo pathway, a key growth control and tumor suppressor pathway originally identified by genetic studies in *Drosophila melanogaster* (for review see Pan, 2010; Halder and Johnson, 2011; Yu et al., 2015). The Hippo pathway is a kinase cascade in which the mammalian homologs of Hippo, the Mammalian Sterile 20-like kinases 1 and 2 (Mst1/2) activate the large tumor suppressor kinases 1 and 2 (LATS1/2), which in turn phosphorylate YAP/TAZ, thereby inducing their cytoplasmic sequestration by 14-3-3 binding and/or leading to ubiquitin-dependent degradation (Figure 2). In the absence of Hippo pathway signaling activity, YAP and TAZ translocate to the nucleus where they serve as transcriptional co-activators that binds predominantly to TEAD family transcription factors (TEAD1-4) and promote target gene expression (Yu et al., 2015; Pan, 2010; Halder and Johnson, 2011). YAP/TAZ/TEAD regulated target genes encode proteins involved in cell proliferation and inhibition of apoptosis (for review see Johnson and Halder, 2014). Thereby, Hippo signaling regulates the balance between cell proliferation, apoptosis, and differentiation to control stem cell proliferation, organ size, as well as tissue homeostasis (Johnson and Halder, 2014). Pathologically, loss of Hippo signaling activity and hyperactivation of its downstream effectors YAP and TAZ through mutations or epigenetic regulation lead to tissue overgrowth and cancer development (Johnson and Halder, 2014).

The Hippo pathway is primarily regulated by mechanical inputs from the microenvironment (Figure 2), including cell-cell contact, matrix stiffness and mechanical stress (stretch or contraction) (for review see Dupont et al., 2011; Meng et al., 2016). In addition, Hippo pathway activity is modulated both, positively and negatively, by G-protein coupled receptors (GPCRs), select cytokine receptors, such as leukemia inhibitory factor receptor, and crosstalk with specific signaling pathways, such as the EGF, Wnt, and bone morphogenic protein (BMP) pathways (Figure 2, Pan, 2010; Halder and Johnson, 2011; Yu et al., 2015; Meng et al., 2016). Mechanical cues are transmitted through activation of cell surface receptors, such as E-cadherins, integrins, and components of the cell polarity complex, as well as certain GPCRs that act as mechanoreceptors (Dupont et al., 2011; Meng et al., 2016). These receptors converge on RhoA and RhoA-dependent regulation of F-actin polymerization and actin-myosin contractility (Dupont et al., 2011; Meng et al., 2016). Cytoskeletal reorganization and contraction, in turn, alter the activity of the cytoplasmic core components of the Hippo pathway leading to phosphorylation-induced cytoplasmic retention and degradation of YAP/TAZ (Dupont et al., 2011; Meng et al., 2016). F-actin architecture is also directly linked to YAP regulation via the Hippo pathway component angiomotin (AMOT) and the related AMOTL1 and AMOTL2 (Chan et al., 2011; Zhao et al., 2011). AMOT family proteins act as adaptors that recruit YAP/TAZ to various cellular compartments, such as cell-cell junctions and F-actin fibers, depending on cellular context (Chan et al., 2011; Dupont et al., 2011; Zhao et al., 2011). Disruption of junction complexes or depolymerization of F-actin releases YAP/TAZ from sequestration by AMOT and drives YAP/TAZ to the nucleus (Chan et al., 2011; Dupont et al., 2011; Zhao et al., 2011). Thus, Hippo pathway activity transforms mechanical signals from the environment through cytoskeletal reorganization into changes in nuclear expression by controlling YAP/TAZ stability and nucleocytoplasmic shuttling. Accordingly, extracellular matrix stiffness affects YAP/TAZ activity and localization, with active YAP/TAZ localizing predominantly in the nucleus in cells grown on a stiff surface, whereas YAP/TAZ remain inactive in the cytoplasm in cells grown on a soft surface (Dupont et al., 2011).

In the mammalian liver, Hippo pathway signaling is involved in development and regeneration as well as pathological processes, such as cancer and fibrosis. During liver development, YAP is important for bile duct formation (tubulogenesis), although the molecular mechanism remains to be elucidated; no effect has been observed from YAP deletion in the adult liver to date (Zhang et al., 2010; Nguyen et al., 2015). However, YAP plays a positive role in the regeneration response following bile duct ligation. Accordingly, nuclear localization of YAP increases after bile duct ligation in mice, and knockout of YAP delays hepatocyte proliferation 5 days post-injury and inhibits biliary epithelial cell proliferation 15 days post-injury (Bai et al., 2012). Conversely, YAP hyperactivation through epigenetic regulation and amplification is common in liver cancer and a wide variety of other cancers, thereby promoting tumorigenesis through cell proliferation, survival and maintenance of the tumor stem cell phenotype (Johnson and Halder, 2014).

YAP is expressed in the hepatocytes of cholestatic livers in young patients (Anakk et al., 2013) as well as in patients with primary sclerosing cholangitis and primary biliary cirrhosis (Bai et al., 2012). Liver diseases often affect the levels of serum bile acids and a recent study reported a direct association of elevated bile acid levels with YAP activation and proliferation of liver progenitor cells, eventually leading to the development of spontaneous liver tumors (Anakk et al., 2013). Importantly, YAP activation is also an early event in HSC activation and fibrosis development. An elegant study by Mannaerts et al., recently demonstrated that YAP drives HSC activation during hepatocellular injury (CCl_4_) induced fibrosis in mice (Mannaerts et al., 2015). The authors showed that YAP translocates into the nuclei of HSCs after CCl_4_ administration, and inhibition of YAP through knockdown or pharmacological inhibition with verteporfin (VP) prevents HSC activation *in vitro* and fibrogenesis *in vivo* (Mannaerts et al., 2015). Knockdown studies and pharmacological inhibition of upstream Hippo pathway components further show that YAP stability and nuclear localization during HSC activation is regulated through modulation of Hippo pathway activity (Figure 2, Mannaerts et al., 2015). The activation of HSC correlates with a switch in gene expression that involves upregulation of genes, which participate in matrix remodeling, actin cytoskeleton, cell proliferation, and immune processes. Importantly, some of the most strongly upregulated genes, such as *Ankrd1* (cardiac ankyrin repeat protein) and *Ctgf* (connective tissue growth factor), are direct targets of YAP (Figure 2, Mannaerts et al., 2015). Ankrd1 is a mechanosensitive transcription factor that is the most highly expressed in the heart and muscle and mediates TGF-β signaling in response to injury and stress (Kojic et al., 2011), whereas CTGF is a cysteine-rich extracellular matrix protein that plays a central role in tissue remodeling and wound repair by regulating the induction of genes, such as fibronectin, collagens (types I, III, IV, and VI) and binding to various cell surface receptors, including αVβ3 and α5β1 integrins (Lau, 2016). Notably, CTGF promotes HSC activation and proliferation and plays a critical role in fibrotic disease (Gressner and Gressner, 2008; Jun and Lau, 2011; Huang and Brigstock, 2012). Significantly, *Ankrd1* and *Ctgf* were upregulated much earlier than *Acta2* (Mannaerts et al., 2015), a widely used marker of HSC activation, consistent with the idea that YAP controls a very early stage in HSC activation. Similar to CCl_4_ treatment, YAP protein and mRNA expression is increased in the livers of mice 5 days after bile duct ligation, and inhibition of YAP through liver-specific conditional deletion reduced bile duct and hepatocyte proliferation, and enhanced hepatocyte necrosis, likely through a Survivin-dependent mechanism (Bai et al., 2012). Thus, YAP regulates the early stages of both, acute, injury-induced, and chronic, cholestatic HSC activation.

During the fibrotic response, YAP serves as a mechanotransduction mediator, integrating signaling responses to mechanical cues to promote proliferation, survival, and extracellular matrix deposition (Dupont et al., 2011; Mannaerts et al., 2015). HSC activation-mediated extracellular matrix stiffening leads to YAP activation, which, in turn, leads to additional extracellular matrix deposition and matrix stiffness ultimately resulting in a feedback loop promoting sustained HSC activation and proliferation (Figure 2, Mannaerts et al., 2015). This was shown through correlative studies in mice and, more directly, using a novel 3D in spheroids system that accurately mimics HSCs activation *in vitro* (Mannaerts et al., 2015). Activation of HSCs by CCl_4_ administration to mice induced nuclear translocation of YAP, increased total YAP protein, and increased picrosirius red (collagen), α-SMA, and ankyrin repeat domain 1 (ANKRD1) staining; this HSC activation was reversed by pharmacologic inhibition of YAP (Mannaerts et al., 2015). Importantly, this process is entirely dependent on matrix stiffness: in 3D cultured, quiescent HSCs with a soft matrix, YAP does not translocate to the nucleus after CCl_4_ administration, but does translocate into the nucleus after CCl_4_ administration to HSCs spread on tissue culture plastic—that is, a stiff matrix—leading to HSC activation as measured by *Acta2, Ankrd1*, and *Ctgf* expression (Butcher et al., 2009; Lu et al., 2012; Mannaerts et al., 2015). Overall, these studies suggest that YAP activates initial steps of HSC activation, thus inhibition of YAP could be a new way for the prevention of liver fibrosis or reverse its progression.

The role of YAP in mechanotransduction during the fibrotic response extends beyond hepatic tissues. Indeed, YAP is expressed in fibrotic, but not healthy, lung tissue (Liu et al., 2015). As in HSCs, when pulmonary fibroblasts are grown on a stiff matrix *in vitro*, YAP accumulates in the nucleus; likewise, knockdown of YAP impedes matrix deposition, contraction and proliferation independent of TGF-β signaling. YAP induces plasminogen activator inhibitor-1 (PAI1), promoting cell-matrix adhesion and continued nuclear accumulation of YAP, thus resulting in a positive feedback loop by which YAP mechanotransduction leads to chronic fibrogenesis (Liu et al., 2015). In contrast to the lung, YAP promotes TGF-β signaling in fibrotic kidneys and, similar to its role in human embryonic stem cells, regulates TGF-β signaling by retaining active SMAD2/3 in the nucleus (Varelas et al., 2008, 2010; Beyer et al., 2013). Similarly to both the liver and the lung, however, YAP promotes renal fibrogenesis by serving as a mechanotransducer. YAP-regulated TGF-β-induced profibrotic SMAD2/3 signaling is inhibited by a soft matrix, but enhanced by a stiff matrix: pharmacologic inhibition of YAP with VP inhibited TGF-β-induced SMAD2/3 signaling *in vitro* in rat kidney fibroblasts as well as the progression of renal fibrosis *in vivo* in a murine unilateral ureteral obstruction model (Szeto et al., 2016). In this study, VP reduced the absolute mRNA levels of YAP and its cofactor transcriptional coactivator with PDZ-binding motif (TAZ) *in vitro*, which is notable because VP is thought to inhibit YAP through direct binding, thereby inducing a conformational change that inhibits interactions with its essential cofactors, TEAD1-4 (Liu-Chittenden et al., 2012). Collectively, these studies strongly implicate Hippo signaling, through YAP, as a critical regulator of chronic fibroproliferative diseases through its mechanotransductive abilities.

## Transcriptional regulation by BRD4 promotes liver fibrosis

Another molecule that plays a key role in regulating profibrotic mechanotransduction at the transcriptional level is the bromodomain and extra terminal domain (BET) family member BRD4. BRD4 is a double bromodomain-containing transcriptional enhancer that binds to acetylated chromatin during interphase and mitosis, thereby serving as a co-activator for transcription of MYC, E2F, and nuclear factor-κB (NF-κB) regulated cytokines to regulate the cell cycle by promoting G2 to M phase transition (reviewed in Shi and Vakoc, 2014; Wang and Filippakopoulos, 2015).

**Figure 3:**
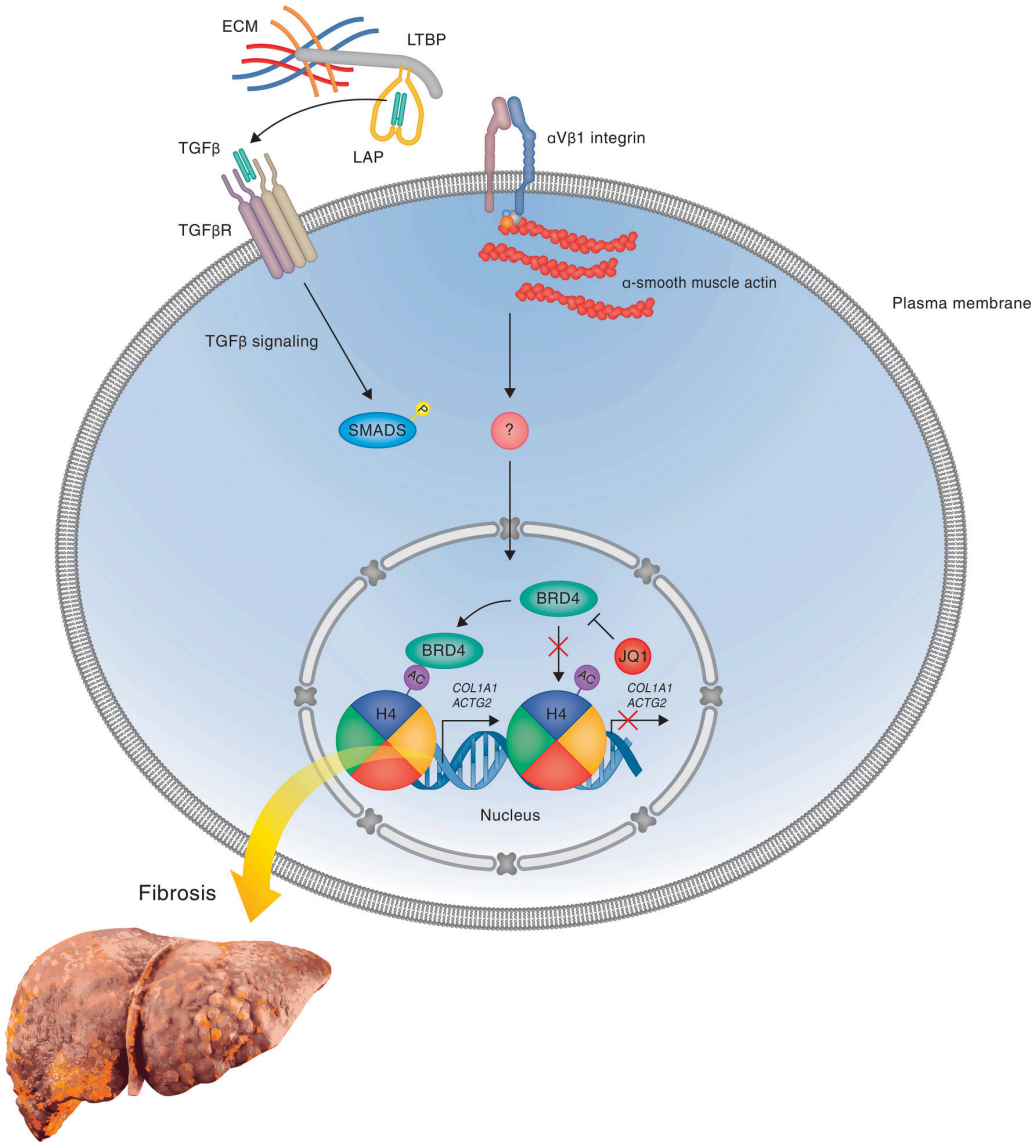
**BRD4 mediated gene expression in liver fibrosis**. A hypothetical model is shown illustrating the regulation of profibrotic gene expression by the double bromodomain-containing transcriptional enhancer BRD4 during liver fibrosis in response to mechanotransduction and attenuation of gene expression by the bromodomain inhibitor JQ1 that prevents the binding of BRD4 to acetylated chromatin. During the fibrotic response, BRD4 binds to acetylated chromatin via its bromodomains and thereby promotes the expression of genes, including *Col1A1*, that are involved in HSC activation, proliferation and matrix remodeling. In the presence of competitive BRD inhibitors, such as JQ1, I-BET (not shown), and PFI-1 (not shown), BRD4 is prevented from binding to acetylated histones, leading to a block in transcription elongation and inhibition of TGF-β-stimulated profibrotic gene expression, thereby attenuating HSC activation and fibrosis development. The precise mechanisms leading to activation of BRD4-mediated transcriptional elongation in response to activation of profibrotic signaling pathways remains to be determined. One potential mechanism involving latent TGF-β activation by integrins is shown.

During the fibrotic response, BRD4 promotes the expression of target genes that are involved in matrix remodeling and proliferation (Figure 3). Of particular note, BRD4 promotes the expression of *Col1A1*, which encodes Type I collagen, in response to exogenous TGF-β, thereby leading to both lung and liver fibrosis (Friedman, 2008; Mederacke et al., 2013; Tang et al., 2013; Ding et al., 2015). Other prominently induced genes encode components of the cytoskeletal apparatus including *Actg2* and *Acta2*, which encode smooth muscle gamma-actin and alpha-actin, respectively (Ding et al., 2015). Inhibition of BRD4 by both siRNA and JQ1 (or the structurally distinct, but functionally similar, chemical BRD inhibitors I-BET-151 and PFI-1) markedly reduced profibrotic mRNA expression, particularly that of *Col1A1* and *Acta2* (Figure 3), inhibited PDGF-mediated HSC proliferation, and blocked HSC activation during CCl_4_-induced liver fibrosis in mice (Ding et al., 2015). Similarly, pharmacologic inhibition of BRD4 with JQ1 reverses bleomycin-induced lung fibrosis in mice, including reduced tissue hydroxyproline and Collagen I staining, indicating that BRD4 inhibition can reduce tissue stiffness-associated profibrotic signaling (Tang et al., 2013).

Collagen is known to activate profibrotic responses in a matrix stiffness-dependent manner, including the formation of stress fibers by increasing *Acta2* (α-SMA) expression and promoting profibrotic TGF-β signaling by applying force to collagen-binding integrin receptors leading to downstream mechanotransduction and crosstalk with SMAD proteins (Kanta, 2015). Furthermore, inhibiting αvβ1 integrin attenuates bleomycin-induced lung and CCl_4_-induced liver fibrosis in mice by inhibiting the processing of latent to active TGF-β (Reed et al., 2015). Thus, collagen deposition leading to integrin activation and mechanotransduction represents a feedback loop promoting pathologic extracellular matrix deposition and sustained profibrotic responses. Considering that BRD4 regulates TGF-β-mediated collagen deposition during the fibrotic response, an attractive mechanistic hypothesis is that BRD4 exacerbates TGF-β-mediated fibrosis and tissue stiffness by increasing signaling through integrin-mediated mechanotransductive pathways, leading to pathologic extracellular matrix remodeling, enhanced tissue stiffness and sustained HSC activation (Figure 3).

## Targeting matrix stiffness-induced gene expression to treat liver fibrosis

While YAP and BRD4 appear to regulate the expression of distinct target genes and therefore likely represent two independent pathways with unique molecular mechanisms, each is regulated by the microenvironment, and each promotes extracellular matrix stiffness and mechanotransductive signaling during liver fibrosis by serving as a coactivator for profibrotic gene transcription. Likewise, each represents a novel target in the treatment of liver fibrosis that can be inhibited by targeting protein-protein interactions with co-activators via small molecules. YAP transcriptional activity can be inhibited with VP. Originally developed as a photosensitizer that is used in FDA-approved photocoagulation therapy for macular degeneration, VP has been found to bind to YAP and inhibit its interactions with TEAD proteins, which are necessary for its effects on transcriptional activation (Liu-Chittenden et al., 2012). VP has been demonstrated to block YAP-induced liver tumorigenesis and suppress hepatomegaly caused by hepatocyte and biliary cell proliferation in YAP transgenic and NF2 knockout mice (Liu-Chittenden et al., 2012; Nguyen et al., 2015). Furthermore, VP blocks matrix stiffness-induced HSC activation *in vitro* as well as CCl_4_-induced fibrogenesis *in vivo*, including a reduction in *Col1A1* gene expression (Mannaerts et al., 2015). As YAP is known to be expressed in human fibrotic liver and lung tissues (Liu et al., 2015; Mannaerts et al., 2015), whether pharmacologic inhibition of YAP by VP can provide a therapeutic effect for patients with fibrosis is an attractive area for further study.

Similarly to the mechanism by which VP inhibits YAP, the competitive bromodomain inhibitor JQ1 inhibits binding between Histone H4 and bomodomains, thereby preventing BRD4 from acting as a transcriptional co-activator of target genes (reviewed in Filippakopoulos and Knapp, 2014). JQ1 was originally developed as an antitumor drug via a small molecule screen, and has proven effective in treating *in vivo* models of acute myeloid leukemia, multiple myeloma and nuclear protein of the testis (NUT) midline carcinomas (reviewed in Filippakopoulos and Knapp, 2014; Wang and Filippakopoulos, 2015). In the context of fibrosis, inhibiting BRD4 with JQ1 inhibits both lung and liver fibrosis as well as HSC activation in murine models, including reducing TGF-β-mediated *Col1A1* expression and extracellular matrix remodeling (Tang et al., 2013; Ding et al., 2015). Thus, JQ1 represents an attractive small molecule for further study as potential treatment of human fibroproliferative diseases and the future development of more specific and stable BRD4 inhibitors (Theodoulou et al., 2016; Waring et al., 2016) may open new opportunities for anti-fibrotic therapy.

## Conclusions and future prospects

The activation of quiescent HSCs and their transition to a proliferative, contractile myofibroblast phenotype represents a critical control point between a normal and pathological tissue repair response. Few regulatory factors have been identified that regulate this transition in the liver and that can be targeted for liver fibrosis reversal. Recent progress has shown that mechanotransduction induced by increased contact between activated hepatic myofibroblasts due to pathologic accumulation of extracellular matrix components and increased focal adhesion formation is orchestrated, in part, by the transcriptional co-activators YAP and BRD4 in concert with their respective transcription factors. Traditionally, small molecule inhibitor development has focused on disrupting ligand-receptor interactions rather than transcriptional regulation. However, no therapies have been developed to date that can reverse liver fibrosis in patients, which has become a major clinical challenge in the industrialized world. More effective therapies are therefore critically needed to treat chronic fibroproliferative diseases. Given that VP and JQ1 have shown efficacy in *in vivo* models of liver fibrosis, targeting the fibrotic response at the transcriptional level presents an attractive avenue for further research.

BRD4 and YAP both regulate profibrotic gene expression that plays a central role in HSC activation, tissue remodeling and liver fibrosis. Nevertheless, these nuclear co-factors appear to regulate distinct sets of target genes suggesting that BRD4 and YAP represent independent pathways that are both critically involved in the regulation of early stages of fibrosis. Thus, there appear to be multiple positive feedback loops involving integrins, TGF-β, and other cytokines that contribute to HSC activation and fibrosis progression by regulating specific nuclear gene expression programs through YAP and BRD4. The studies by Mannaerts et al. (2015) and Ding et al. (2015) suggest that by inhibiting either YAP or BRD4 action, these positive feedback loops can be broken, leading to attenuation or reversal of fibrosis. If true, the rational combinations of YAP and BRD4 inhibitors may provide synergistic effects and offer a more effective way to reverse fibrosis progression than the single agents and this should be a further avenue of research. Toward this aim, it will be important to confirm the roles of YAP and BRD4 in fibrosis development and reversal using inducible knockout mouse mutants for YAP and BRD4 and to evaluate in more detail the anti-fibrotic action and efficacy of VP and JQ1 in disease relevant animal models. In addition, a more detailed understanding of the regulation of and interaction between YAP and BRD4 pathways will be necessary to define the precise roles of these pathways in HSC activation and fibrosis. While YAP and BRD4 each appear to control different populations of target genes, it is likely that the two pathways cooperate during HSC activation. Indeed, a recent study using the BRD inhibitor JQ1 revealed a role of BET bromodomain protein(s) in regulating the activity of the YAP homolog TAZ (Duan et al., 2016) indicating a possible point of cross-talk between the two profibrotic pathways.

Last, but not least, of particular interest will be the elucidation of the precise mechanisms by which mechanical signals from the environment are relayed across the membrane to modulate YAP and BRD4 function in the nucleus. Integrins are the primary mechanosensors and transducers of tissue stiffness and several integrins have been identified as key drivers of profibrotic signaling in various organs. Some of these integrins, in particular αv subunit integrins, such as αvβ1 and αvβ6, have been shown to control the activation of latent TGF-β1 complex and targeting these integrins with small molecule inhibitors can block or reverse fibrosis progression in mouse models (Henderson and Sheppard, 2013; Henderson et al., 2013; Reed et al., 2015; Conroy et al., 2016). Given that BRD4 is critical for TGF-β-dependent profibrotic gene expression, BRD4 activity may be one of the downstream targets of integrin and TGF-β action.

Alternatively, integrins can control HSC activation through bi-directional “outside-in” and “inside-out” signaling mechanisms leading to changes in actin organization and actomyosin contractility and subsequent alterations in tissue remodeling. Indeed, several integrin-associated signaling proteins, including integrin-linked kinase (ILK, Zhang et al., 2006; Li et al., 2009; Kavvadas et al., 2010; Shafiei and Rockey, 2012; Radovanac et al., 2013), focal adhesion kinase (FAK, Wong et al., 2011; Lagares et al., 2012; Kinoshita et al., 2013; Lagares and Kapoor, 2013; Zhao et al., 2016), and regulators and effectors of Rho GTPases, such as P-Rex1 (Liang et al., 2016), PAK and Rho-activated kinase (ROCK, Knipe et al., 2015; Martin et al., 2016) have been identified as potential targets for anti-fibrotic therapy in a number of organs. Notably, activation of ILK, which links F-actin to focal adhesions and is required for integrin-mediated force generation, sustains nuclear YAP accumulation in pulmonary arterial vascular smooth muscle cells to promote cell proliferation in pulmonary arterial hypertension, a disease characterized by increased deposition of extracellular matrix and vascular stiffness (Kudryashova et al., 2016). Consistent with this notion, regulation of Hippo activation by ILK (Serrano et al., 2013), Src-FAK (Kim and Gumbiner, 2015; Li et al., 2016), and beta1 integrins (Martin et al., 2016) has been observed in the context of epithelial-mesenchyme transition, cancer, as well as tissue homeostasis in the skin (Elbediwy et al., 2016). Although the physiological relevance of Hippo pathway regulation by integrins and their effectors needs to be confirmed in the context of fibrosis there is accumulating evidence supporting such a role. Indeed, while this review was under consideration, the integrin α11β1 was reported to promote liver fibrosis by modulating hepatic stellate cell activation through the Hippo/YAP pathway (Martin et al., 2016). Interestingly, knockdown of the α11 subunit in HSCs by RNA interference abolished a subset of YAP-regulated genes during HSC activation and partially reversed tissue fibrosis in a mouse model of the disease, indicating that this particular integrin represents one of likely multiple mechanosensitive upstream regulators of YAP in liver fibrosis (Martin et al., 2016).

In sum, mechanotransduction in myofibroblasts is clearly relevant for fibrotic disease and it is increasingly being realized that components of mechanosensitive signal transduction pathways are not only drivers of disease progression, but also promising targets for therapy. While additional work will be required to address the precise roles of YAP and BRD4 in fibrosis, overall the current findings suggest that YAP and BRD4 are key terminal effectors of mechanosensing signal transduction pathways that initiate and perpetuate fibrosis and that targeting YAP and BRD4 alone or in combination has great promise toward treating patients with organ fibrosis and reducing the burden of fibroproliferative diseases. A more refined understanding of the mechanisms underlying myofibroblast activation across different organ systems and better insight into the roles and regulation of YAP and BRD4 in this process could lead to more personalized treatment options that may slow and perhaps even reverse fibrosis progression in the future.

## Author contributions

AZ, AT, KK, LW, and JK have written this review. All authors have read the review and gave their agreement for submission.

### Conflict of interest statement

The authors declare that the research was conducted in the absence of any commercial or financial relationships that could be construed as a potential conflict of interest.
